# Antibody levels to tetanus, diphtheria, measles and varicella in patients with primary immunodeficiency undergoing intravenous immunoglobulin therapy: a prospective study

**DOI:** 10.1186/1471-2172-15-26

**Published:** 2014-06-21

**Authors:** Fernanda Aimée Nobre, Isabela Garrido da Silva Gonzalez, Raquel Maria Simão, Maria Isabel de Moraes Pinto, Beatriz Tavares Costa-Carvalho

**Affiliations:** 1 Federal University of Sao Paulo, Rua dos Otonis, 731, CEP: 04025-002 São Paulo, Brazil

**Keywords:** Immunoglobulins, Intravenous, Antibody deficiency syndromes, Tetanus, Diphtheria, Measles, Chickenpox

## Abstract

**Background:**

Patients with antibody deficiencies depend on the presence of a variety of antibody specificities in intravenous immunoglobulin (IVIG) to ensure continued protection against pathogens. Few studies have examined levels of antibodies to specific pathogens in IVIG preparations and little is known about the specific antibody levels in patients under regular IVIG treatment. The current study determined the range of antibodies to tetanus, diphtheria, measles and varicella in IVIG products and the levels of these antibodies in patients undergoing IVIG treatment.

**Methods:**

We selected 21 patients with primary antibody deficiencies who were receiving regular therapy with IVIG. Over a period of one year, we collected four blood samples from each patient (every 3 months), immediately before immunoglobulin infusion. We also collected samples from the IVIG preparation the patients received the month prior to blood collection. Antibody levels to tetanus, diphtheria, measles and varicella virus were measured in plasma and IVIG samples. Total IgG levels were determined in plasma samples.

**Results:**

Antibody levels to tetanus, diphtheria, varicella virus and measles showed considerable variation in different IVIG lots, but they were similar when compared between commercial preparations. All patients presented with protective levels of antibodies specific for tetanus, measles and varicella. Some patients had suboptimal diphtheria antibody levels. There was a significant correlation between serum and IVIG antibodies to all pathogens, except tetanus. There was a significant correlation between diphtheria and varicella antibodies with total IgG levels, but there was no significant correlation with antibodies to tetanus or measles.

**Conclusions:**

The study confirmed the variation in specific antibody levels between batches of the same brand of IVIG. Apart from the most common infections to which these patients are susceptible, health care providers must be aware of other vaccine preventable diseases, which still exist globally.

## Background

Intravenous immunoglobulin (IVIG) is a therapeutic preparation containing pooled antibodies (IgG) from blood and plasma donors. One of the main areas of IVIG application is as antibody replacement therapy in patients with quantitative or qualitative antibody deficiencies. These patients critically depend on the presence of a variety of antibody specificities in IVIG to ensure continued protection against any viral or microbial pathogens they might encounter. The broad spectrum of antimicrobial activities in these preparations is crucial for reducing infections [1,2].

Many factors may have an impact on the quality and quantity of antibodies in immunoglobulin products [2,3]. Differences in some specific titres between commercially available products have been shown [3-5]. Moreover, nowadays, for some diseases, plasma donor immunity is conferred by vaccination and not by natural infection and studies have demonstrated an association between vaccine-induced immunity and a decrease in specific antibody levels to some diseases [6-8].

Despite the importance of IVIG in conferring protection, few studies have examined levels of antibodies to specific pathogens in IVIG preparations and little is known about the specific antibody levels in patients with antibody deficiency under regular IVIG treatment. In replacement therapy it is important that patients receive protective levels of antibodies to infections that are preventable by vaccines and, also, to common pathogens that cause infections in patients with antibody deficiencies.

The objective of the current study was to determine the range of antibodies to some bacterial and viral pathogens in IVIG products and also the levels of these antibodies in patients undergoing IVIG treatment, over a one-year period.

## Results

We selected 21 patients with a mean age of 25 years old, 11 male, with the following diagnosis: six with X-linked Agammaglobulinemia (XLA), twelve with Common Variable Immunodeficiency (CVID) and three with Hyper IgM Syndrome (HIM), who were undergoing regular IVIG replacement therapy every 4 weeks. The hallmark of these immunodeficiency diseases is a severely impaired IgG production. The mean IgG at diagnosis was 226 mg/dL (range 5–564,6 mg/dL; normal values in adults: 739–1390 mg/dL). The mean IgG level during the study was 778 mg/dL (range 459–1220 mg/dL) and the mean IVIG dose was 553 mg/kg/month (range 340–760 mg/kg/month). Over the study, the IVIG dose remained unchanged for each patient. Most patients received more than one IVIG commercial preparation during the study, because they depend on the preparation provided by the government.

Thirty-eight lots of six different commercial IVIG preparations and eighty-four plasma samples were evaluated.

### Antibody levels in IVIG preparations

Antibody levels to tetanus, diphtheria, varicella and measles showed considerable variation in each of the 38 different lots (Table 1). For all antigens tested, the coefficient of variation was greater than 50% (Table 1).

**Table 1:** Pathogen-specific antibody levels in IVIG preparations

**IVIG**	**Mean IU/mL**	**SD**	**Number of lots**
**Tetanus**	**19,92**	**10,19**	**38**
Immunoglobulin**®**	20,04	1,60	2
Endobulin**®**	9,25	1,45	3
Flebogamma**®**	26,02	10,64	6
Octagam**®**	18,99	7,91	16
Tegeline**®**	18,64	13,68	9
Vigam**®**	30,58	3,70	2
**Diphtheria**	**10,97**	**9,58**	**38**
Immunoglobulin**®**	12,87	6,97	2
Endobulin**®**	7,82	5,29	3
Flebogamma**®**	19,86	19,35	6
Octagam**®**	10,05	6,48	16
Tegeline**®**	7,75	4,11	9
Vigam**®**	8,98	5,41	2
**Measles**	**28,53**	**18,53**	**38**
Immunoglobulin**®**	43,15	41,56	2
Endobulin**®**	9,65	0,53	3
Flebogamma**®**	35,04	12,73	6
Octagam**®**	23,04	16,10	16
Tegeline**®**	39,06	18,76	9
Vigam**®**	19,04	6,98	2
**Varicella**	**21,75**	**12,19**	**38**
Immunoglobulin**®**	19,47	10,87	2
Endobulin**®**	11,06	7,88	3
Flebogamma**®**	32,26	11,30	6
Octagam**®**	21,60	13,28	16
Tegeline**®**	20,90	10,12	9
Vigam**®**	13,62	0,42	2

The bold data represent the mean for all of the 38 different lots.

The titres of antibodies were compared between commercial preparations of Flebogamma®, Octagam® and Tegeline®. Titres to tetanus, diphtheria, varicella and measles were similar for these three products (tetanus*: p = 0.051*; diphtheria: *p = 0.254*; varicella: *p = 0.615*; measles: *p = 0.588*). Immunoglobulin®, Endobulin® and Vigam® were administered only to a few patients so we did not have sufficient numbers of lots for comparison (Table 1).

### IgG and antibody levels in serum samples

IgG and antibody levels did not remain constant during the follow up. There was considerable variation in specific antibody and total IgG levels throughout the year in these patients (Table 2).

**Table 2:** IgG and antibody levels in serum samples for each of the four samplings

	**Mean**	**SD**	**Min**	**Max**	**n**
**Tetanus (IU/mL)**					
**First sample**	0,82^(A)^	0,28	0,34	1,57	21
**Second sample**	1,72^(B)^	0,77	0,79	3,48	21
**Third sample**	1,82^(B)^	0,81	0,16	3,25	21
**Fourth sample**	2,22^(B)^	2,22	0,17	9,90	21
**Diphtheria (IU/mL)**					
**First sample**	0,49^(C)^	0,25	0,07	0,96	21
**Second sample**	0,61^(C)^	0,38	0,14	1,53	21
**Third sample**	0,64^(C)^	0,46	0,08	1,65	21
**Fourth sample**	1,22^(D)^	0,53	0,16	2,15	21
**Measles (IU/mL)**					
**First sample**	2,21^(E)^	0,67	0,81	3,47	21
**Second sample**	2,88^(E)^	1,83	0,86	6,82	21
**Third sample**	1,74^(F)^	0,99	0,60	4,30	21
**Fourth sample**	2,66^(E)^	1,51	1,09	7,94	21
**Varicella (IU/mL)**					
**First sample**	0,86^(G)^	0,84	0,27	4,06	21
**Second sample**	1,45^(H)^	0,60	0,70	2,57	21
**Third sample**	1,50^(H)^	0,61	0,48	2,67	21
**Fourth sample**	2,33^(I)^	1,18	0,60	5,27	21
**Total IgG (mg/dL) – plasma samples**					
**First sample**	748,71^(J)^	167,26	459,0	1040,0	21
**Second sample**	741,76^(J)^	154,01	521,0	1030,0	21
**Third sample**	754,19^(J)^	162,62	535,0	1070,0	21
**Fourth sample**	868,62^(K)^	162,85	634,0	1220,0	21

^(A)^and ^(B)^have different mean at a significant level of 5% (p = 0.017).

^(C)^and ^(D)^have different mean at a significant level of 5% (p < 0.001).

^(E)^and ^(F)^have different mean at a significant level of 5% (p = 0.043).

^(G)^,^(H)^ and ^(I)^have different mean at a significant level of 5% (p < 0.001).

^(J)^and ^(K)^have different mean at a significant level of 5% (p = 0.0156).

All patients presented protective levels of tetanus, measles and varicella antibodies. There were patients with suboptimal diphtheria antibody levels (Table 2 - minimum values).

### Correlation of serum specific antibodies with IVIG specific antibodies

**Figure 1:**
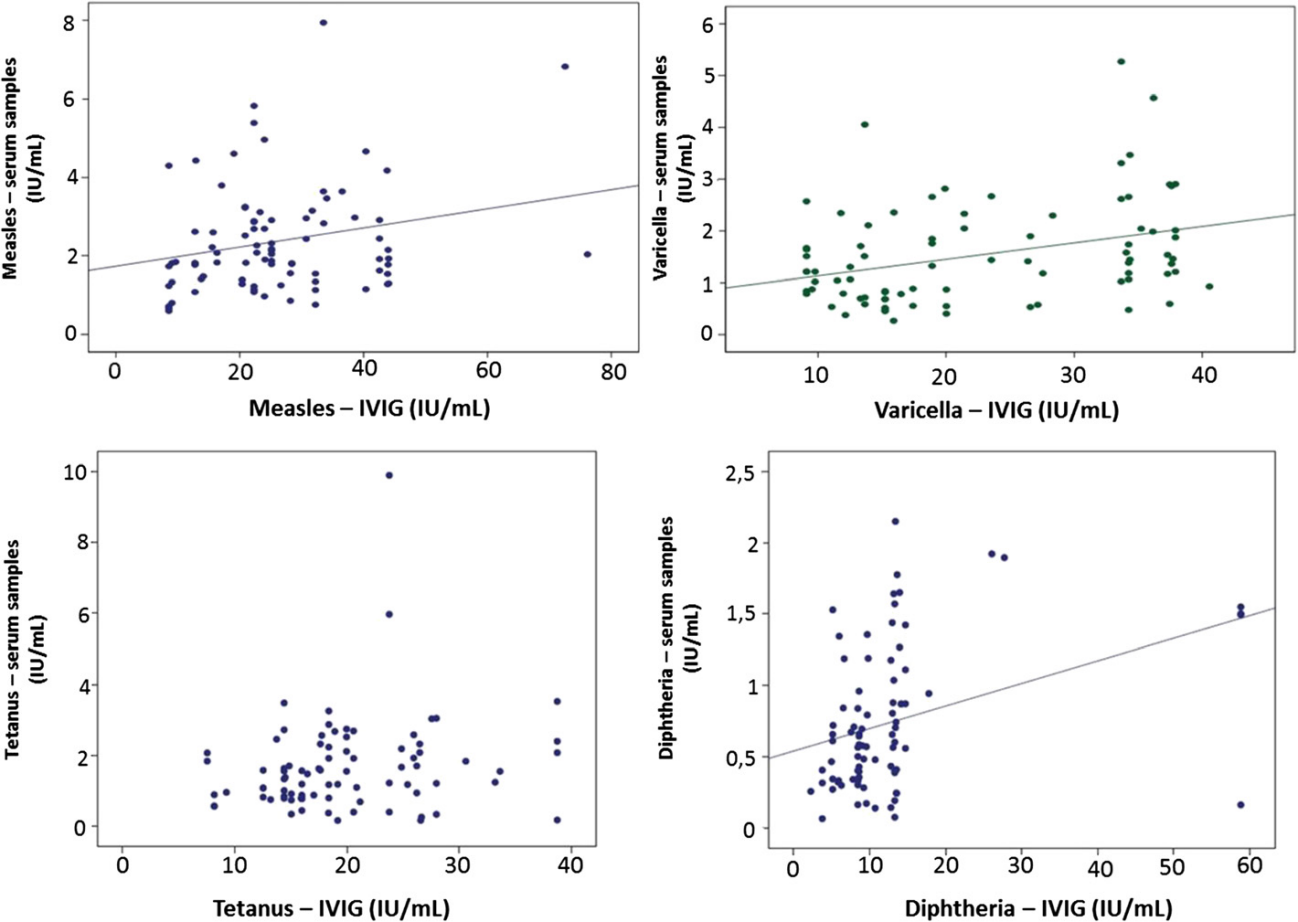
**Correlation between serum and IVIG antibodies.** Measles: r = 0.232; p = 0.034. Varicella: r = 0.342; p = 0.001. Tetanus: r = 0.189; p = 0.084. Diphtheria: r = 0.355; p = 0.001.

There was significant correlation between serum and IVIG antibodies to all pathogens, except tetanus (Figure 1).

### Correlation of pathogen-specific antibodies with total IgG levels

**Figure 2:**
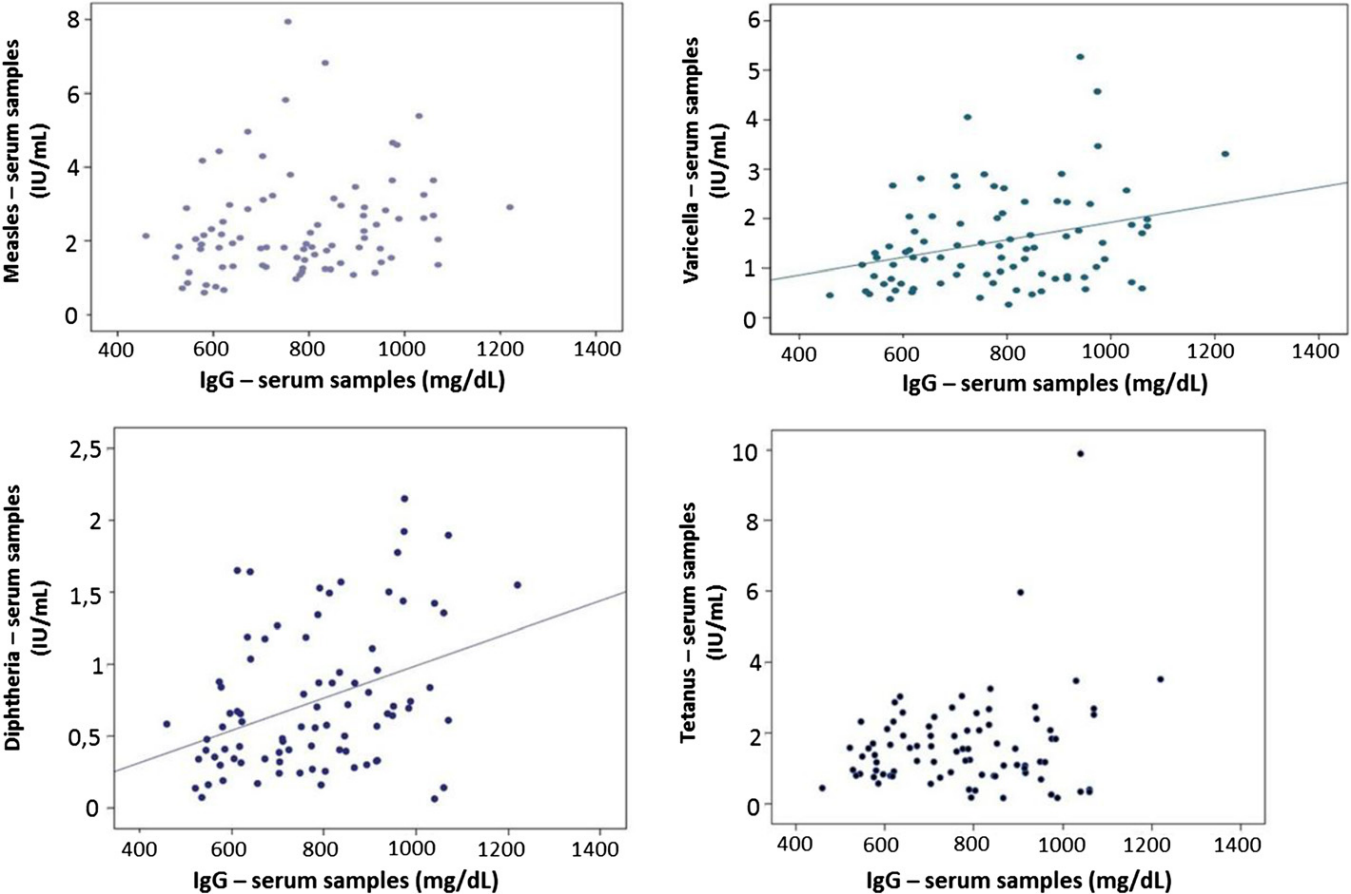
**Correlation between pathogen-specific antibodies with total IgG levels.** Measles: r = 0.210; p = 0.056. Varicella: r = 0.302; p = 0.005. Tetanus: r = 0.209; p = 0.057. Diphtheria: r = 0.377; p = 0.0004.

There was a significant correlation between diphtheria and varicella antibodies with total IgG levels, but there was no significant correlation to tetanus or measles (Figure 2).

Antibody levels to tetanus, diphtheria, varicella and measles were similar between patients with XLA and patients with CVID and HIM. (tetanus: *p = 0.680*; diphtheria: *p = 0.221*; varicella: *p = 0.73*; measles: *p = 0.360*).

Antibody levels to tetanus, varicella and measles were similar between patients receiving IVIG doses equal or greater than 500 mg/kg and patients receiving doses smaller than 500 mg/kg (tetanus: *p = 0.665*; varicella*: p = 0.140*; measles: *p = 0.592*). Antibody levels to diphtheria were greater in patients receiving IVIG doses greater than 500 mg/kg (*p = 0.031*).

## Discussion

Recent studies have shown that for some microorganisms there is significant variation in antibody levels in IVIG preparations of different brands, as well as in different batches of the same brand [5-7]. For measles, varicella, tetanus and diphtheria, there was no significant difference in antibody concentrations between the three different commercial preparations of IVIG. However, there is variability in the levels of these antibodies among different batches of the same brand, which probably correlates to plasma donor’s antibody levels.

Several studies have shown the efficacy of IVIG treatment in reducing the number and severity of infections in patients with humoral deficiency [9-13]. Most studies, however, focus their attention on the ability of the treatment to reduce the number of respiratory infections, since this is the main cause of death in this population [9-13]. Little is discussed in relation to protection against less common but potentially serious infectious diseases. During this study, one patient with diagnosis of XLA, in regular use of IVIG, developed mild varicella zoster infection, despite adequate levels of specific antibodies against varicella (2,03 IU/mL) on first day of infection. Serum levels of specific antibodies against various pathogens have been associated with protection. Some studies have shown that individuals with specific serum antibody levels do not develop disease after exposure to some pathogen [14]. However, these protective levels are usually determined after active immunization in people who can produce antibodies. There are no known studies that outline what the minimum serum antibody levels against measles, varicella, tetanus or diphtheria should be that can ensure protection by passive immunization for patients who do not produce their own antibodies.

We expected that all patients regularly using adequate doses of IVIG would have protective antibody levels against these diseases. However, two patients had diphtheria antibody concentrations below the recommended protective levels. Although it was only observed on one occasion, these patients susceptibility to diphtheria was worrying, as this disease has not yet been eradicated here in Brazil. The diphtheria antibody concentration in the patients’s serum may be associated, among other factors, with the levels of these antibodies in IVIG preparations used, as we observed a significant positive linear correlation between the specific serum antibody concentration from each patient and the samples of administered IVIG to these patients the previous month. Antibody levels for vaccine preventable disease, such as measles, have been reduced in IVIG in recent years due to the increased number of vaccinated donors and the reduction of wild-type pathogen circulating [6].

The effective dosing of IVIG in patients with antibody deficiencies is determined by their ability to control infections and the residual total IgG level (collected immediately prior to IVIG infusion) [1,2]. In these patients, specific antibody levels to several pathogens are rarely measured. There is no consensus on the ideal dose to be administered or the ideal total IgG level maintained by the patient on regular use of IVIG. Nowadays it is highly recommended that the IVIG dose, as well as the serum total IgG target should be analyzed individually [10,15-17]. However, some patients with satisfactory total IgG levels develop infections against pathogens which they should be theoretically protected against by IVIG. Recent studies have hypothesized that even while maintaining an adequate level of total IgG, some patients may not have sufficient levels of specific antibodies to various pathogens [10,17]. For diphtheria and varicella, we observed a positive linear correlation between total IgG serum concentration and specific antibodies, however, one patient with diphtheria antibody levels below the recommended protective level, had a total IgG concentration greater than 1g/dL. For measles and tetanus, there was no significant correlation between the total IgG serum concentration and specific antibody concentrations in the patients. We suggest that a satisfactory level of total IgG does not necessarily mean adequate levels of specific antibodies in the same individual, for some pathogens.

## Conclusions

Our study assumed that the heterogeneity of the immune status of the potential plasma donor from the healthy population may have implications on specific antibody levels in immunoglobulin preparations and our study also strengthened the variation in specific antibody concentrations between batches of the same brand of IVIG. Apart from the most common infections to which these patients are susceptible, health care providers must be aware of other vaccine preventable diseases, which still exist globally.

## Methods

We conducted this trial at the Federal University of São Paulo - Brazil. The study protocol was approved by the Ethics Committee at the University and a written informed consent was obtained from each enrolled patient or from his/her parents.

We selected 21 patients with primary antibody deficiencies: X-linked Agammaglobulinemia (XLA), Common Variable Immunodeficiency (CVID) and Hyper IgM syndrome (HIM), who were under regular replacement therapy with IVIG (every 4 weeks) for a minimum of two years. Patients with protein loss disorders were excluded from the study. Over a period of one year, from 2009 to 2010, we collected 4 blood samples from each of these patients (every 3 months), immediately before immunoglobulin infusion. We also collected samples from the IVIG that these patients had received the month prior to blood collection. Antibody levels to tetanus, diphtheria, measles and varicella were determined in plasma and IVIG samples. Total IgG levels were determined only in plasma samples.

Diphtheria and tetanus IgG antibodies were measured by an in-house double-antigen ELISA and measles and varicella IgG antibodies were measured by an in-house indirect ELISA:

• Double antigen ELISA to detect tetanus antibodies: Tetanus toxoid (Butantan Institute) diluted in 0.1M carbonate-bicarbonate buffer, pH 9.6, was used to coat 96-well microtiter plates overnight at 4°C. Two-fold serial dilutions of plasma samples and of tetanus reference serum (in-house standard calibrated against “Tetanus antitoxin human immunoglobulin NIBSC reagent 1976 (76/589)”) in dilution buffer (10 mM PBS, pH 7.2, 1% Triton X-100) with 1% bovine serum albumin were added to the plate and incubated for 1 h at 37°C. Biotin-labeled tetanus toxoid in dilution buffer was then added to the plate and incubated for 1 h at 37°C. Streptavidin-alkaline phosphatase conjugate (Zymed, San Francisco, CA, USA) in dilution buffer was incubated for 1 h at 37°C. p-Nitrophenyl-phosphate (Sigma, St. Louis, MO, USA) in 1M diethanolamine, 5 mM magnesium chloride buffer, pH 9.8, was used as substrate and absorbance at 450 nm was read with an immunoreader ELX-800 (Bio-Tek Instruments, Winooski, VT, USA). Between steps, the plate was washed five times in dilution buffer. Tetanus antibodies are reported as IU/mL using the curve comparison method to transform optical density to concentration units.

• Double antigen ELISA to detect diphtheria antibodies [18]. The same method was used for diphtheria antibodies, with some modifications: diphtheria toxoid (Butantan Institute), diphtheria reference serum (in-house standard calibrated against “Diphtheria antitoxin human serum 91/534” - NIBSC reagent) and biotin-labeled diphtheria toxoid were used. Diphtheria antibodies are reported as IU/mL using the curve comparison method to transform optical density to concentration units.

• Indirect ELISA to detect VZV antibodies [19]. Varicella IgG antibodies were assessed by an “in house” indirect ELISA. MaxiSorp 96-well microtiter plates (Nunc, New York, EUA) were coated with varicella vaccine (Varilrix, SmithKline Beecham, Belgium) diluted 1:100 in 0.1M carbonate-bicarbonate buffer, pH 9.6, and incubated overnight at 4°C. Two-fold serial dilutions of plasma samples and of varicella reference serum [in house standard calibrated against “Varicella zoster virus antibody human immunoglobulin-NIBSC reagent (90/690)”] in 0.01M phosphate buffered saline (PBS), pH 7.2 and 0.05% Tween 20 with 1% bovine serum albumin (BSA) were added to the plate and incubated for 1 h at 37°C. Reference serum was added to 10 wells and serum samples were added to 3 wells, in all twofold dilutions starting at 1:100. In the next step, alkaline phosphatase conjugated rabbit anti-human IgG, specific for γ-chains (Invitrogen, USA) diluted 1:500 in 0.01M PBS, pH 7.2 and 0.05% Tween 20, was incubated for 1 h at 37°C. pnitrophenyl-phosphate disodium (Sigma, USA) in 0.1M diethanolamine, 0.005M magnesium chloride buffer, pH 9.8, was used as substrate in a concentration of 1 mg/mL. OD was read at 405 nm in an immunoreader ELX-800, using 630 nm as a reference filter (Bio-Tek Instruments, USA). Between steps, the plate was washed five times in 0.01M PBS, pH 7.2 and 0.05% Tween 20. All solutions were added in a 100 μL volume to microplate wells. Varicella zoster antibodies were expressed in IU/mL using the curve comparison method to transform optical density in concentration units. In all plates two blank wells we always present, and mean values were subtracted from all other wells.

• Indirect ELISA to detect measles antibodies: Measles IgG antibodies were assessed by an “in house” indirect ELISA. MaxiSorp 96-well microtiter plates (Nunc, New York, EUA) were coated with measles antigen (cell supernatant of measles-infected cells) (Microbix, Toronto, Canada) or control measles antigen (cell supernatant of non-infected cells) (Microbix) diluted 1:100 in 0.1M carbonate-bicarbonate buffer, pH 9.6, and incubated overnight at 4°C. Two-fold serial dilutions of plasma samples and of measles reference serum [in house standard calibrated against the WHO International Standard Anti-Measles Serum (NIBSC code: 66/202) in 0.01M phosphate buffered saline (PBS), pH 7.2 and 0.05% Tween 20 with 1% bovine serum albumin (BSA) were added to the plate and incubated for 1 h at 37°C. Reference serum was added to 10 wells and plasma samples were added to 3 wells, in all twofold dilutions starting at 1:100. In the next step, alkaline phosphatase conjugated rabbit anti-human IgG, specific for γ-chains (Invitrogen, USA) diluted 1:500 in 0.01M PBS, pH 7.2 and 0.05% Tween 20, was incubated for 1 h at 37°C. p-Nitrophenyl-phosphate disodium (Sigma, USA) in 0.1M diethanolamine, 0.005M magnesium chloride buffer, pH 9.8, was used as substrate in a concentration of 1 mg/mL. OD was read at 405 nm in an immunoreader ELX-800, using 630 nm as a reference filter (Bio-Tek Instruments, USA). Between steps, the plate was washed five times in 0.01M PBS, pH 7.2 and 0.05% Tween 20. All solutions were added in a 100 μL volume to microplate wells. Measles antibodies were expressed in IU/mL using the curve comparison method to transform optical density in concentration units. OD values from wells coated with control antigen were subtracted from OD values from wells coated with measles antigen.

According to the levels established for the healthy population, tetanus and diphtheria antibody levels equal to or greater than 0.1 IU/mL were considered to be fully protective, antibody levels equal to or greater than 0.01 and below 0.1 IU/mL were considered with basic immunity and antibody levels below 0.01 IU/mL were considered nonimmune [19]. Measles antibody levels equal to or greater than 0.12 IU/mL were considered protective [20]. Varicella antibody levels equal to or greater than 0.1 IU/mL were considered to be fully protective, antibody levels equal to or greater than 0.05 and below 0.1 IU/mL were considered with basic immunity and antibody levels below 0.05 IU/mL were considered nonimmune [21].

### Statistical analysis

Mean, corresponding SDs, median, maximum and minimum and CV were used to summarize values. The following tests were used to analyse the IVIG and plasma antibody content: student’s t test, ANOVA and Kruskal-Wallis. Correlation was analysed assuming Gaussian distribution (Pearson correlation). A P value of < 0.05 was considered significant.

## Abbreviations

ANOVA: Analysis of variance; CV: Coefficient of variation; ELISA: Enzyme-linked immunosorbent assay; IVIG: Intravenous immunoglobulin; SD: Standard deviation.

## Competing interests

The authors declare that they have no competing interests.

## Authors’ contributions

FAN and IGSG have made substantial contributions to conception and design, acquisition of data and analysis and interpretations of data. RMS carried out the immunoassays. BTCC and MIMP have been involved in revising the manuscript critically for important intellectual content and have given the final approval of the version to be published. All authors have read and approved the final manuscript.

## Authors’ information

Fernanda Aimée Nobre and Isabela Garrido da Silva Gonzalez, The two first authors made equal contributions. The authors certify that each had a first author role equally.
